# The Book World of Medicine and Science

**Published:** 1899-12-09

**Authors:** 


### THE BOOK WORLD OF MEDICINE AND SCIENCE.

Transactions of the Jenner Institute of Preventive Medicine. Second series. (London: Macmillan and Co. 1899.)

This volume commences with a description of the new building at Chelsea, from which it will be seen how extensive is the accommodation provided. There are a board room, waiting room, and offices; a private office for the director, with two laboratories, one for himself and one for his assistant, which together will afford space for six workers; a general bacteriological laboratory for twenty-five students, in connection with which is a small operating room; rooms for the preparation of culture media, and a room which will be kept at incubator temperature; rooms for electro-motors and general workshops, and a cold storage installation. There is also a photographic department, one of the two photomicrographic cameras being fitted with the electric arc light. Then there is a chemical laboratory for twenty students; the Hansen laboratory for the study of the practical application of bacteriology to industrial and technical processes; a series of rooms have also been fitted up for private research; and there are the laboratories and office at present used by the Local Government Board for the preparation and storage of glycerinated calf lymph. Besides all these there are a lecture theatre, a museum, a large animal house, and a crematorium. This outline of the accommodation will suffice to show how complete is the institution which is now at the disposal of those who are willing to devote their energies to investigations bearing on the prevention of disease. The rest of the volume is occupied by a series of papers which, with one exception, are descriptive of work done in the laboratories of the institute. The exception is an article by Professor Erhlich on the constitution of the diphtheria bacillus. Naturally the papers are of a highly technical character. We congratulate the Jenner Institute on being able to make such a show of good work so soon after getting possession of its new premises.

Matter, Ether, and Motion: The Factors and Relations of Physical Science. By A. E. Dolbear, Ph.D. English edition. Edited by Professor Alfred Lodge. (London: Society for Promoting Christian Knowledge. 1899. Price 5s.)

Professor Dolbear has shown a considerable amount of boldness in attempting the task of explaining in the ordinary language of common life the abstruse principles of physical science. Yet the event has justified the attempt. After carefully reading this volume we can recommend it, not merely as giving a most instructive and suggestive outline of our present knowledge of the subjects of which it treats, but as drawing back the screen and exposing to the view of the student some of those larger conceptions and wider theories by which, in the minds of the leaders, the facts of physical science are linked together. The book commences with a description of matter and its properties, and this is followed by an account of ether, in which the line is drawn smartly and distinctly between ether and all forms of matter, and the student is introduced to the mystery of vortex rings, on the understanding of which so much depends. Then come chapters on motion, energy, gravitation, and heat; after which one on ether waves, chiefly illustrated by reference to light, introduces us to other manifestations of physical energy, such as electricity, chemism, and sound. There are very useful chapters on physical fields, on machines and mechanism, and, finally, on the properties of matter. From the above outline it will be seen how wide a view is taken of the whole subject, and if the book be read it will also be seen how these different branches of physical science are made to interlock, and how clearly their inter-dependence is demonstrated. This, in fact, is the strong part of the book—the argument by which it is held together. At the same time it must be stated that the merely descriptive parts, such as those dealing with sound, light, and electricity, are very clear. Take it altogether we should describe the work as an admirable introduction to the study of physical science. At the same time it may be doubted whether it is entirely the sort of book on which the ordinary cut-and-dried examinations, which mostly work to a syllabus rather than to broader generalisations, could best be passed. Fortunately there are still some left who have not bent the knee to the examination bogie, and for such this is just the book.

### NOTES ON BOOKS.

A little pamphlet entitled "Common-Sense Health Reform," by T. Thatcher, among other questions deals with that of open windows. All sorts of people nowadays are advocating open windows. Where the common-sense, however, comes in is in Mr. Thatcher's insistence on the wearing of appropriate apparel, and maintaining the exposure to the open air continuously. "In the depth of winter," he says, "with rain or snow beating into my room, I still keep my window wide open, and am always well, seldom take cold, have good appetite, am cheerful, ruddy as a hunter, and ever ready for my work, and, with a good walk once or twice a week, I get, perhaps, as much fresh air as most men who lead outdoor lives." We certainly congratulate Mr. Thatcher on his "condition."

The Christmas number of "Cassell's Magazine" includes an admirable photogravure of "The Fortune Teller," which was exhibited in the Academy this year.

The subject of diseases of the nails is so imperfectly treated in most text-books on skin diseases that it may be well to draw attention to the admirable articles on this subject which have appeared in recent numbers of the "Archives of Medicine," by Mr. Jonathan Hutchinson, F.R.S. The articles are illustrated with many very well-executed coloured plates, besides woodcuts in the text.

A little book which we have already favourably noticed is "A Synopsis of the British Pharmacopœia, 1898," compiled by H. Whippell Gadd, with analytical tables and suggested standards by C. G. Moor, M.A., public analyst for the City of Exeter (Baillière, Tindall, and Cox; price 1s.) This has now achieved a fourth edition, and undoubtedly it deserves its success, for it has skimmed the cream off the Pharmacopœia and has served it up in such a form as to give in the smallest possible compass almost all of it that the prescriber generally wants to know.

The Annual Report of the Howard Association is an interesting little pamphlet, which shows what an amount of useful work may be done at the expenditure of a comparatively small sum of money when affairs are directed by an energetic secretary such as this association possesses in Mr. Tallack. Naturally there is not much to show as the direct outcome of what has been done, but no one who reads the report can doubt that the existence of such an organisation as this, which has for its aim the throwing of light into all the dark spots of prison life, is of very great service to humanity.

